# Association of maternal characteristics with latino youth health insurance disparities in the United States: a generalized structural equation modeling approach

**DOI:** 10.1186/s12889-020-09188-1

**Published:** 2020-07-11

**Authors:** Cinthya K. Alberto, Jessie Kemmick Pintor, Brent Langellier, Loni Philip Tabb, Ana P. Martínez-Donate, Jim P. Stimpson

**Affiliations:** 1 Dornsife School of Public Health, Drexel University, 3600 Market St, Philadelphia, PA 19104 USA; 2 Department of Health Management and Policy, Dornsife School of Public Health, Drexel University, 3215 Market St, Nesbitt Hall, Philadelphia, PA 19104 USA; 3 Department of Epidemiology and Biostatistics, Dornsife School of Public Health, Drexel University, 3215 Market St, Nesbitt Hall, Philadelphia, PA 19104 USA; 4 Department of Community Health and Prevention, Dornsife School of Public Health, Drexel University, 3215 Market St, Nesbitt Hall, Philadelphia, PA 19104 USA

**Keywords:** Maternal child health, Access to health care, Health insurance, Immigration, Latinos, Disparities, Ethnicity

## Abstract

**Background:**

Disparities in access to care persist for Latino youth born in the United States (US). The association of maternal characteristics, such as maternal citizenship status and insurance coverage, on youth health insurance coverage is unclear and is important to examine given the recent sociopolitical shifts occurring in the US.

**Methods:**

We analyzed pooled cross-sectional data from the 2010–2018 National Health Interview Survey to examine the association of Latina maternal citizenship status on maternal insurance coverage status and youth uninsurance among US-born Latino youth. Our study sample consisted of 15,912 US-born Latino youth (ages < 18) with linked mothers. Our outcome measures were maternal insurance coverage type and youth uninsurance and primary predictor was maternal citizenship status. Generalized structural equation modeling was used to examine the relationships between maternal characteristics (maternal citizenship, maternal insurance coverage status) and youth uninsurance.

**Results:**

Overall, 7% of US-born Latino youth were uninsured. Just 6% of youth with US-born mothers were uninsured compared to almost 10% of those with noncitizen mothers. Over 18% of youth with uninsured mothers were uninsured compared to 2.2% among youth with mothers who had private insurance coverage. Compared to both US-born and naturalized citizen Latina mothers, noncitizen Latina mothers had 4.75 times the odds of reporting being uninsured. Once adjusted for predisposing, enabling, and need factors, maternal uninsurance was strongly associated with youth uninsurance and maternal citizenship was weakly associated with youth uninsurance among US-born Latino youth.

**Conclusion:**

Maternal citizenship was associated with both maternal uninsurance and youth uninsurance among US-born Latino youth. Federal- and state-level health policymaking should apply a two-generational approach to ensure that mothers of children are offered affordable health insurance coverage, regardless of their citizenship status, thus reducing uninsurance among US-born Latino youth.

## Background

Latino youth in the United States (US) face persistent disparities in access to and utilization of health care services compared to their non-Latino white counterparts. These disparities may be related, in part, to parental immigration status [1, 2]. Half of all Latino youth in the US have at least one immigrant parent and approximately 4 million have at least one parent who is undocumented [3]. US-born Latino youth with immigrant parents are more likely to be uninsured and to have discontinuous health insurance coverage and are less likely to have private insurance compared to US-born Latino youth with US-born parents [4–7]. Immigrant parents are also more likely to lack awareness of and experience with health insurance, more likely to experience contextual restrictions on eligibility, and less likely to work for employers who offer health insurance [8]. Access to programs and eligibility, such as Medicaid, varies by citizenship status and can indirectly hinder access to health care services for US-born youth in immigrant families [9, 10].

Immigration and citizenship status have been the primary predisposing factor to understand the association between parental characteristics and youth health care access outcomes. One study found a positive association between maternal citizenship and Latino youth health insurance coverage [11], and found that even among insured immigrant parents, youth with parents who were noncitizens were more likely to be uninsured [12–14], Medicaid and Children’s Health Insurance Program (CHIP) eligible youth with citizen parents were more likely to have higher participation rates compared to youth with noncitizen parents [15], Latina immigrant mothers seeking access to health services for their US-born youth may experience discrimination based on predisposing and/or enabling factors (e.g., limited English language proficiency, citizenship status) and may experience obstacles in obtaining necessary documents (e.g., proof of income) [16].

Parental enabling factors have been found to be associated with youth health insurance coverage. State-level Medicaid expansion and insuring parents with public insurance was associated with higher levels of youth public insurance coverage, retention, and continuity [17, 18]. For Latino populations in the US, sociopolitical factors have increasingly become a concern for health care access and utilization. For instance, an increase in risk of deportation is associated with a decrease in Medicaid use among mothers of youth in mixed status families [19]. Inclusive and exclusionary, federal- and state-level immigration and health policies, have implications for the health and well-being of immigrants and their youth in the US [20, 21]. For instance, Latina mothers who are not US citizens and work for employers who violate labor laws and who do not offer benefits may be more likely to be uninsured [22], which could make it more likely that their US-born Latino youth may also be uninsured and further decrease their youth’s likelihoods of receiving preventive pediatric care and annual well-child visits [23]. Little is known on the association between maternal citizenship status and maternal insurance coverage status and their association with Latino youth uninsurance.

Recent anti-immigrant and anti-Latino rhetoric [24] and public charge regulation changes [25] make it timely and important to understand the pathways via which maternal characteristics, such as citizenship status and insurance coverage status, are associated with Latino youth health care access in the US. Consistent with other studies and with our aim to study the associations between predisposing, enabling, and need factors on youth health care access, we used the Andersen Behavioral Model of Health Services [26] as a framework to understand the direct and indirect pathways between maternal citizenship, maternal insurance coverage status, and youth uninsurance. We hypothesize that maternal citizenship and insurance coverage will be directly and indirectly associated with youth uninsurance among US-born Latino youth. Specifically, maternal insurance coverage status will be associated with youth uninsurance, and maternal citizenship status will be directly associated with maternal insurance coverage status and with youth insurance coverage status.

## Methods

We used Stata 15 for all analyses [27] and relied on pooled 2010–2018 National Health Interview Survey (NHIS) data supplied by the Integrated Public Use Microdata Series (IPUMS) [28]. IPUMS NHIS allows for parental characteristics (e.g., maternal age, maternal educational attainment) to be linked to youth observations thus allowing for the analysis of youth linked to their mothers’ characteristics. NHIS is a nationally representative, cross sectional household interview survey of the civilian, non-institutionalized population in the US. NHIS is an annual survey administered by the National Center for Health Statistics and provides information on the health of the population and covers topics such as insurance coverage, use of medical services, and health behaviors. The annual response rate of NHIS is approximately 70% of eligible households in the US [29]. Our study sample consists of US-born Latino youth less than 18 years of age with linked Latina maternal characteristics, such as foreign-born status (born in the United States or US territory, born outside of the United States), citizenship status (citizen, noncitizen), and insurance coverage status (private insurance coverage, public insurance coverage, uninsured). Latina mothers included in the sample were less than 65 years of age and who reported that their youngest youth was no older than 18 years old at the time of the survey (*N* = 15,912).

Our outcome measure of youth health care access was maternal reports on whether their youth had insurance coverage for the past year (uninsurance). The main predictors were maternal reports of maternal nativity and citizenship status (US-born citizen, naturalized citizen, noncitizen) and maternal insurance coverage status (private insurance coverage no/yes, public insurance coverage no/yes, uninsured no/yes). Control variables included predisposing characteristics of maternal marital status (married, divorced/separated, never married), interview language (English/English and Spanish, Spanish/Other), maternal education level (less than high school degree, high school degree, college degree or more), maternal employment status (working, with a job but not at work), regional residence (Northeast, North Central/Midwest, South, West), and maternal age (18–29, 30–39, 40–49, and 50–64 years). Enabling factors included maternal income as a percent of the federal poverty level (FPL) (400% and above, 200–399, 199% and below). Need factors included maternal reports of their own general health status and of their youth’s general health status (excellent or very good, good, fair or poor).

Sample descriptive statistics were performed via analysis of variance (ANOVA) tests to examine whether there were sample population mean differences in predisposing, enabling, need, and contextual variables within maternal nativity and citizenship status and maternal insurance coverage status groups. We performed a path analysis to understand the hypothesized pathways between maternal characteristics and youth uninsurance [30]. Generalized structural equation models (GSEM) were used to test the various hypotheses, where logit GSEMs were utilized due to the binary nature of our outcomes of interest. The path analysis consisted of three logit models simultaneously estimated to examine: (1) the direct association of maternal citizenship and maternal predisposing, enabling, and need characteristics on maternal insurance coverage status, (2) the association of maternal insurance coverage status on youth uninsurance, and (3) maternal characteristics, particularly maternal citizenship status, on youth uninsurance. Akaike’s information criterion were included in all GSEM models to show model fit. All models were adjusted for sample weights and complex survey design for findings to represent the noninstitutionalized Latino youth population in the US, and survey year. Probit GSEM were calculated and compared with the logit model estimates to ensure the results were not due to sensitivity to model specification.

## Results

Table 1 shows descriptive sample characteristics of US-born Latino youth and Latina maternal characteristics by maternal citizenship status and by maternal insurance coverage status. Almost 40% of all youth in our sample had mothers reporting that they were noncitizens, and around 34% of all youth in our sample had mothers who reported being uninsured. Overall, almost 8% of youth were uninsured with variation existing within maternal citizenship status and maternal insurance coverage status. Slightly over 6% of youth with US-born mothers were uninsured compared to almost 10% of those with noncitizen mothers. Approximately 2.2% of youth with moms who had private insurance coverage were uninsured compared to over 18% of youth with moms who were uninsured. US-born citizen mothers and mothers with private insurance coverage were more likely to report incomes above 200% FPL compared to noncitizen and uninsured mothers. Noncitizen and uninsured mothers were more likely to have less than a high school degree compared to US-born citizen and privately insured mothers.

**Table 1:** Sample characteristics among US-born Latino youth and Latina mothers by maternal foreign-born and citizenship status, and maternal insurance coverage status

	Total	Maternal Citizenship Status				Maternal Insurance Coverage Status			
		US-born Citizen	Naturalized Citizen	Noncitizen		Private	Public	Uninsured	
**N**	15,912	7154	2517	6241	*(P)*	6173	4284	5455	*(P)*
**Weighted %**	100	44.9	15.8	39.2		38.8	26.9	34.3	
**Youth Uninsurance**									
No	92.4	93.7	93.1	90.6	< 0.001	97.8	99.1	81.1	< 0.001
Yes	7.6	6.3	6.9	9.4		2.2	0.9	18.9	
**Predisposing Factors**									
Maternal Marital Status									
Married	63.3	55.1	75.4	67.9	< 0.001	75.7	44.8	61.9	< 0.001
Divorced/Separated	13.1	14.8	15.5	10.1		11.3	17.2	12.4	
Never Married	23.6	30.1	9.1	22.0		13.0	38.0	25.7	
Interview Language									
English/ Spanish and English	77.5	95.6	81.0	55.4	< 0.001	90.4	76.4	63.5	< 0.001
Spanish/Other	22.5	4.4	19.0	44.6		9.7	23.6	36.5	
Maternal Education Level									
Less than a High School Degree	32.0	14.0	20.7	57.1	< 0.001	12.2	37.2	49.9	< 0.001
High School Degree	53.6	66.5	57.6	37.2		57.5	57.9	45.4	
College Degree or more	14.4	19.5	21.7	5.8		30.3	4.9	4.7	
Maternal Employment									
Working/With a job	58.1	66.7	68.9	43.9	< 0.001	78.3	45.4	45.7	< 0.001
Unemployed	6.0	6.4	5.3	5.8		2.1	9.9	7.7	
Not in Labor Force	35.9	26.9	25.8	50.3		19.7	44.7	46.6	
Maternal Age									
18–29	31.0	39.3	16.2	27.5	< 0.001	20.3	42.1	33.4	< 0.001
30–39	46.3	41.9	46.2	51.4		49.2	40.1	48.9	
40–49	19.2	15.9	30.8	18.4		25.5	15.0	15.9	
50–64	3.4	2.9	6.9	2.7		4.9	2.9	1.9	
US Census Region									
Northeast	12.5	11.8	18.3	10.9	0.953	12.2	22.4	8.4	0.462
North Central/Midwest	10.0	10.5	8.1	10.3		10.8	9.7	9.3	
South	37.5	36.2	36.5	39.3		38.3	20.3	50.2	
West	40.0	41.5	37.2	39.5		38.8	47.7	32.1	
**Enabling Factor**									
Income (%FPL)									
200% and above	38.5	51.0	50.8	19.2	< 0.001	73.1	14.6	17.1	< 0.001
100–199%	31.8	26.4	30.7	38.4		21.5	36.0	39.6	
99% and below	29.7	22.6	18.4	42.4		5.4	49.4	43.3	
**Need Factors**									
Maternal General Health Status									
Excellent/very good	60.7	62.4	65.8	56.8	< 0.001	69.2	52.6	56.9	< 0.001
Good	30.5	28.3	27.5	34.3		25.1	34.7	33.3	
Fair/poor	8.8	9.3	6.8	8.9		5.7	12.7	9.7	
Youth General Health Status									
Excellent/very good	79.4	81.9	81.8	75.5	< 0.001	85.8	75.1	74.8	< 0.001
Good	18.2	15.8	16.1	21.8		13.2	20.9	22.3	
Fair/poor	2.4	2.2	2.1	2.6		1.0	4.1	2.9	
**Contextual Characteristic**									
Year									
2010–2013	43.2	41.7	40.0	46.3	< 0.001	40.2	37.9	50.1	< 0.001
2014–2015	22.8	22.3	23.0	23.4		23.0	24.6	21.5	
2016–2018	33.9	36.1	37.0	30.3		36.7	37.6	27.9	

Weighted column percentages

*FPL* federal poverty level

ANOVA test *(P)* values

IPUMS National Health Interview Survey, 2010–2018

**Fig. 1:**
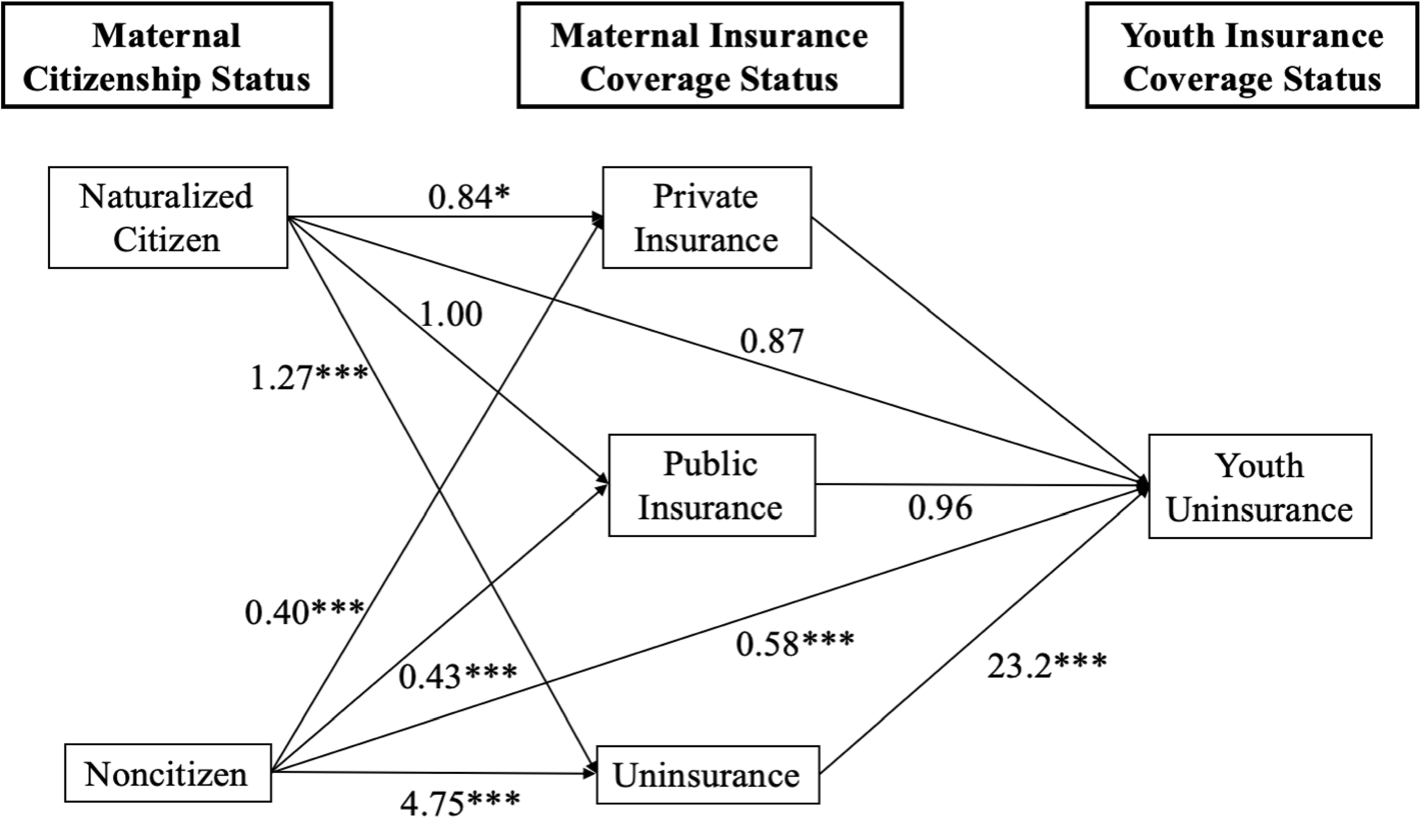
Multivariate adjusted odds ratios of generalized structural equation model logit estimation of the association among maternal citizenship, maternal insurance coverage status and youth uninsurance. Reference groups are youth with US-born citizen mothers and youth with mothers who have private insurance coverage. Estimates are adjusted for maternal marital status, interview language, maternal educational level, maternal employment, maternal age, region, income, maternal general health status, and youth general health status. *P < 0.05. ***P < 0.001

Exponentiated logit coefficients from the GSEM analysis on the relationship between maternal characteristics, maternal insurance coverage status, and youth uninsurance are presented in Fig. 1. Noncitizen Latina mothers were less likely to report having private (OR, 0.40; 95% CI, 0.34–0.45) or public health insurance coverage (OR, 0.43; 95% CI, 0.38–0.49) compared to both US-born citizen mothers. Naturalized citizen mothers were also less likely to report having private insurance coverage compared to US-born citizen mothers (OR, 0.84; 95% CI, 0.73–0.97). Compared to US-born citizen Latina mothers, naturalized citizen Latina mothers were more likely to report being uninsured (OR, 1.27; 95% CI, 1.10–1.47). Noncitizen Latino mothers were more likely to report being uninsured compared to US-born Latina mothers (OR, 4.75; 95% CI, 3.56–4.83).

After adjusting for predisposing, enabling, and need factors, we observed that Latino youth with noncitizen mothers were less likely to be uninsured compared to youth with US-born citizen mothers (OR, 0.58; 95% CI, 0.47–0.72). Youth with uninsured mothers were significantly more likely to be uninsured themselves (OR, 23.2; 95% CI, 17.2–31.1) compared to youth with mothers who had private insurance coverage.

We included an appendix containing full results from the GSEM analysis from Fig. 1 on the relationship among main predictors, control variables, and youth uninsurance (Additional file 1: Table S1) and an abbreviated table of probit GSEM analysis results on the relationship between maternal citizenship, maternal insurance coverage type, and youth insurance coverage status (Additional file 1: Table S2). The probit estimates were similar to the logit estimates.

## Discussion

US-born Latino youth face persistent disparities in access to health care services compared to their non-Latino white counterparts [1, 2]. Given that more than half of all US-born Latino youth have at least one immigrant parent [3], and access to programs vary by citizenship status, parental characteristics may indirectly contribute to health care access disparities for youth in immigrant families [9, 10]. Previous studies have shown an association between maternal citizenship and youth insurance coverage among Latinos [11] and that youth with noncitizen insured parents were more likely to be uninsured [12–14]. The relationship between maternal characteristics and youth health care access is limited in the literature, and to our knowledge, no other study has examined the association of maternal citizenship and insurance coverage status on health insurance coverage for US-born Latino youth. This study observed that being a noncitizen mother is directly related to maternal uninsurance and that maternal uninsurance is positively associated with youth uninsurance. Maternal citizenship status is indirectly associated with youth uninsurance for US-born Latinos. Our findings build on observations from previous studies by estimating the magnitude of the direct relationship between maternal characteristics and youth uninsurance. Elucidating these relationships is important given the present and ongoing anti-immigrant and Latino rhetoric [24] and public charge regulation changes [25] that may increase health care access disparities for US-born Latino youth in immigrant families.

Noncitizen Latina mothers are more likely to be uninsured compared to US-born and naturalized citizen mothers in our study which is consistent with previous reports that found over 20% of lawfully present immigrants and more than 40% of undocumented immigrants are uninsured compared to less than 8% of US citizens [22]. These disparities could be related with citizenship status-based restrictions on eligibility for public insurance, and/or a greater likelihood of noncitizens working for employers who do not offer health insurance and/or who violate labor and wage laws, leading to lower incomes that limit the ability to afford paying premiums for private insurance or employer sponsored insurance [22]. Contextual characteristics are also associated with insurance coverage disparities for Latina immigrant mothers. For example, an increase in risk of deportation was associated with a decrease in Medicaid use among Latina mothers in mixed status families [19]. Lastly, the passage of exclusionary federal- and state-level immigration and health policies, such as the current administration’s changes to public charge policy [25] or undocumented immigrants being excluded from the Patient Protection and Affordable Care Act (ACA), influence health care access and the overall health of immigrants and of their US-born youth [20, 21].

We found that youth with noncitizen mothers were less likely to be uninsured compared to youth with US-born and naturalized citizen mothers. This is contrary to a previous finding that youth with Medicaid and CHIP eligible parents were more likely to be insured compared to youth with noncitizen parents [15]. This finding is a weak association compared to the association between maternal uninsurance and youth uninsurance and thus should be interpreted with caution. Maternal uninsurance is significantly associated with youth uninsurance and maternal citizenship is associated with maternal uninsurance, indicating that there is a strong indirect pathway between maternal citizenship and youth uninsurance. While maternal citizenship was indirectly related to youth uninsurance, we found that youth with noncitizen mothers were less likely to be uninsured but, importantly, that maternal uninsurance was related to youth uninsurance and noncitizen mothers had much higher odds of being uninsured than their citizen counterparts, which is important given that US-born Latino youth make up the largest racial and ethnic group among all youth in the US [31] and many of them are members of immigrant families. This indirect pathway in the relationship between maternal citizenship and youth uninsurance, as we observed, may operate through maternal uninsurance—given that citizenship is used to determine eligibility for full-time employment and health insurance coverage options (e.g., health insurance marketplaces from the ACA [22]), noncitizen mothers are more likely to be uninsured compared to citizen mothers. Maternal citizenship is associated with maternal insurance coverage status, which is then associated with youth uninsurance among US-born Latino youth.

Our findings may vary by region or state in the US. We found that US-born Latino youth who reside in the Southern region of the US had the highest odds of being uninsured nationally, which is consistent with policy restrictions on public benefits for immigrants compared to the Northeast [32]. Latina immigrant mothers may also be more likely to experience discrimination when attempting to access care for their youth based on their own characteristics, such as their English language proficiency and citizenship status [16], which may be more common in the South [33]. If immigrant Latina mothers continue to be excluded from health care access by restrictive federal and state policies [34], then the disparities in health care outcomes among US-born Latino youth could widen.

Having access to health insurance coverage is a significant factor in utilizing services, especially the preventive care and annual well-child visits for youth suggested by the American Academy of Pediatrics [35]. In this study, while we examined health insurance coverage status for youth and their mothers, it is important to discuss that maternal citizenship and its association with insurance is indicative of systemic discrimination based on documentation and citizenship status [10]. Health policies that are enacted in the US and at the state-level can either be inclusive or exclusionary [10] in nature and in this study, we observed that being a noncitizen was strongly associated with being uninsured. This demonstrates that US health policy is excluding noncitizens explicitly from accessing affordable health insurance coverage options.

Our findings should be interpreted within several limitations. First, we cannot determine the relationships between several different variations of citizenship and documentation status (e.g., legal permanent resident, undocumented) and that may lead to underestimating the relationship between maternal citizenship and maternal uninsurance, and youth uninsurance. For instance, if in our noncitizen category, legal permanent residents are overrepresented, we may be underestimating the relationship between maternal citizenship status and maternal insurance for moms who may be undocumented and their youth’s uninsurance. Youth uninsurance may be far greater among youth with mothers who are undocumented and uninsured, and we are unable to capture this level of detail in our citizenship measure. Second, we rely on a repeated cross-sectional design survey and we cannot control whether maternal characteristics change over time per observation even with the use of fixed effects for survey year. For instance, if a mother is a noncitizen and becomes a citizen and we cannot examine how this change would influence their own and their youth’s uninsurance. Third, while we control for region of residence, we were unable to include state level indicators which would be more informative given that state level policies have larger health implications for immigrant populations. Lastly, while our use of generalized structural equation modeling has been novel in our understanding of the pathways between maternal characteristics and youth uninsurance for US-born Latino youth, we cannot fully confirm the direction of causality within the relationships we sought to understand because the data are cross-sectional.

## Conclusion

Maternal characteristics are associated with youth uninsurance among US-born Latino youth. Specifically, maternal citizenship is a pathway to youth uninsurance through maternal insurance coverage status suggesting that ensuring health care access for Latino youth in the US may depend on whether mothers have adequate access to health care. States can improve access to health care by utilizing a two-generational approach to health policy and expand Medicaid and CHIP to all children regardless of their own and family’s immigration status, thus further reducing youth uninsurance for youth in immigrant families.

## Supplementary information

**Additional file 1: Table S1.** Odds ratios from generalized structural equation model logit estimation of Latina maternal characteristics, Latina maternal insurance coverage status, and youth uninsurance. **Table S2.** Odds ratios from generalized structural equation model probit estimation of Latina maternal citizenship status, Latina maternal insurance coverage status, and youth uninsurance.

## Data Availability

The dataset that was analyzed during the study are available for download for free from the Integrated Public Use Microdata Series National Health Interview Survey website (https://nhis.ipums.org/nhis/).

## Abbreviations

US: United States of America
CHIP: Children’s Health Insurance Program
NHIS: National Health Interview Survey
IPUMS: Integrated Public Use Microdata Series
FPL: Federal Poverty Level
ANOVA: Analysis of Variance
GSEM: Generalized Structural Equation Modeling
OR: Odds ratio (i.e. exponentiated logit coefficient)
ACA: Patient Protection and Affordable Care Act
