# Supplementary material for: Association of maternal characteristics with latino youth health insurance disparities in the United States: a generalized structural equation modeling approach
Source: BMC Public Health. 2020 Jul 11;20:1088. doi: 10.1186/s12889-020-09188-1 (PMC7353771; doi:10.1186/s12889-020-09188-1)
Supplement: Supplementary file 1 — Additional file 1: Table S1. Odds ratios from generalized structural equation model logit estimation of Latina maternal characteristics, Latina maternal insurance coverage status, and youth uninsurance. Table S2. Odds ratios from generalized structural equation model probit estimation of Latina maternal citizenship status, Latina maternal insurance coverage status, and youth uninsurance. [file 12889_2020_9188_MOESM1_ESM.docx]

**Appendix:**

| **Table 1: Odds ratios from generalized structural equation model logit estimation of Latina maternal characteristics, Latina maternal insurance coverage status, and youth uninsurance.** | | | | |
| --- | --- | --- | --- | --- |
|  | **Maternal Insurance Coverage Status** | | | **Youth Insurance Coverage Status** |
|  | **Private** | **Public** | **Uninsured** | **Uninsured** |
|  | **Odds Ratio**  **[95% CI]** | **Odds Ratio**  **[95% CI]** | **Odds Ratio**  **[95% CI]** | **Odds Ratio**  **[95% CI]** |
| **Predisposing Factors** |  |  |  |  |
| Maternal Citizenship Status |  |  |  |  |
| US-born Citizen | REF. | REF. | REF. | REF. |
| Naturalized Citizen | 0.84*  [0.73-0.97] | 1.00  [0.87-1.16] | 1.27***  [1.10-1.47] | 0.87  [0.69-1.11] |
| Noncitizen | 0.40***  [0.34-0.45] | 0.43***  [0.38-0.49] | 4.75***  [4.25-5.30] | 0.58***  [0.47-0.72] |
| Maternal Insurance Coverage |  |  |  |  |
| Private |  |  |  | REF. |
| Public |  |  |  | 0.96  [0.62-1.51] |
| Uninsured |  |  |  | 23.2***  [17.2-31.1] |
| Maternal Marital Status |  |  |  |  |
| Married | REF. | REF. | REF. | REF. |
| Divorced/Separated | 0.62***  [0.53-0.72] | 1.84***  [1.60-2.11] | 0.98  [0.85-1.13] | 0.83  [0.65-1.06] |
| Never Married | 0.47***  [0.41-0.54] | 1.89***  [1.68-2.11] | 1.08  [0.97-1.21] | 0.70***  [0.58-0.86] |
| Interview Language |  |  |  |  |
| English or Spanish and English | REF. | REF. | REF. | REF. |
| Spanish or Other | 0.71***  [0.61-0.82] | 1.02  [0.90-1.16] | 1.28***  [1.15-1.44] | 0.92  [0.75-1.12] |
| Maternal Education Level |  |  |  |  |
| Less than High School Degree | REF. | REF. | REF. | REF. |
| High School Degree | 1.62***  [1.43-1.84] | 0.90  [0.80-1.11] | 0.80***  [0.72-0.89] | 0.86  [0.72-1.03] |
| College Degree or more | 3.63***  [3.01-4.37] | 0.45***  [0.36-0.56] | 0.38***  [0.31-0.46] | 0.90  [0.65-1.25] |
| Maternal Employment |  |  |  |  |
| Working/With a job | REF. | REF. | REF. | REF. |
| Unemployed | 0.23***  [0.18-0.30] | 1.76**  [1.47-2.11] | 1.51**  [1.25-1.82] | 1.07  [0.80-1.42] |
| Not in Labor Force | 0.40***  [0.36-0.45] | 1.75***  [1.57-1.95] | 1.19***  [1.08-1.31] | 0.74***  [0.63-0.87] |
| Maternal Age |  |  |  |  |
| 18-29 | REF. | REF. | REF. | REF. |
| 30-39 | 1.80***  [1.59-2.04] | 0.63***  [0.56-0.70] | 0.95  [0.85-1.05] | 1.11  [0.93-1.32] |
| 40-49 | 2.24***  [1.92-2.62] | 0.66***  [0.57-0.76] | 0.73***  [0.63-0.84] | 1.50***  [1.20-1.87] |
| 50-64 | 3.27***  [2.41-4.44] | 0.61***  [0.47-0.81] | 0.45***  [0.33-0.61] | 1.27  [0.81-2.00] |
| US Census Region |  |  |  |  |
| Northeast | REF. | REF. | REF. | REF. |
| North Central/Midwest | 1.90***  [1.53-2.35] | 0.41***  [0.33-0.50] | 1.62**  [1.33-1.97] | 1.47*  [1.04-2.16] |
| South | 1.34***  [1.13-1.59] | 0.19***  [0.16-0.22] | 3.49***  [2.97-4.09] | 1.90***  [1.38-2.62] |
| West | 1.33***  [1.13-1.57] | 0.75***  [0.65-0.86] | 1.23*  [1.05-1.44] | 1.75***  [1.28-2.41] |
| **Enabling Factor** |  |  |  |  |
| Income (%FPL) |  |  |  |  |
| 200% and above | REF. | REF. | REF. | REF. |
| 100-199% | 0.25***  [0.23-0.28] | 3.77***  [3.25-4.36] | 2.43***  [2.14-2.76] | 0.60***  [0.49-0.74] |
| 99% and below | 0.08***  [0.07-0.09] | 6.89***  [5.90-8.05] | 2.62***  [2.29-2.99] | 0.38***  [0.30-0.47] |
| **Need Factor** |  |  |  |  |
| Maternal General Health Status |  |  |  |  |
| Excellent/very good | REF. | REF. | REF. |  |
| Good | 0.87*  [0.78-0.97] | 1.19***  [1.07-1.32] | 1.01  [0.92-1.12] |  |
| Fair/poor | 0.78*  [0.63-0.95] | 1.27**  [1.09-1.49] | 0.99  [0.85-1.17] |  |
| Youth General Health Status |  |  |  |  |
| Excellent/very good |  |  |  | REF. |
| Good |  |  |  | 0.96  [0.79-1.18] |
| Fair/poor |  |  |  | 1.24  [0.78-1.98] |
| **Contextual Characteristic** |  |  |  |  |
| Year |  |  |  |  |
| 2010-2013 | REF. | REF. | REF. | REF. |
| 2014-2015 | 1.08  [0.97-1.22] | 1.55***  [1.40-1.73] | 0.67***  [0.61-0.74] | 0.89  [0.74-1.06] |
| 2016-2018 | 0.92  [0.82-1.04] | 2.00***  [1.78-2.25] | 0.64***  [0.57-0.72] | 0.90  [0.75-1.10] |
|  |  |  |  |  |
| AIC | 3.40e+07 | 3.66e+07 | 3.97e+07 | 1.70e+07 |
| CI, confidence interval; Ref., reference; AIC, Akaike's Information Criterion.  **P*<0.05.  ***P*<0.01.  ****P*<0.001.  *Source*: IPUMS National Health Interview Survey, 2010-2018, N = 15,912 | | | | |

| **Table 2: Odds ratios from generalized structural equation model probit estimation of Latina maternal citizenship status, Latina maternal insurance coverage status, and youth uninsurance.** | | | | |
| --- | --- | --- | --- | --- |
|  | **Maternal Insurance Coverage Status** | | | **Youth Insurance Coverage Status** |
|  | **Private** | **Public** | **Uninsured** | **Uninsured** |
|  | **Odds Ratio**  **[95% CI]** | **Odds Ratio**  **[95% CI]** | **Odds Ratio**  **[95% CI]** | **Odds Ratio**  **[95% CI]** |
| **Predisposing Factors** |  |  |  |  |
|  |  |  |  |  |
| **Model 1** |  |  |  |  |
| Maternal Citizenship Status |  |  |  |  |
| US-born Citizen | REF. | REF. | REF. | REF. |
| Naturalized Citizen | 0.90*  [0.83-0.97] | 1.01  [0.93-1.10] | 1.11*  [1.02-1.21] | 0.95  [0.84-1.07] |
| Noncitizen | 0.58***  [0.54-0.62] | 0.63***  [0.58-0.67] | 2.50***  [2.35-2.67] | 0.76***  [0.68-0.85] |
|  |  |  |  |  |
| **Model 2** |  |  |  |  |
| Maternal Insurance Coverage |  |  |  |  |
| Private |  |  |  | REF. |
| Public |  |  |  | 1.03  [0.86-1.24] |
| Uninsured |  |  |  | 4.53***  [3.94-5.20] |
|  |  |  |  |  |
| AIC | 3.40e+07 | 3.67e+07 | 3.99e+07 | 1.71e+07 |
| Model 1 adjusted for marital status, interview language, maternal educational level, maternal employment, maternal age, region, survey year, income, and maternal general health status.  Model 2 adjusted as model 1 + youth general health status.  CI, confidence interval; Ref., reference; AIC, Akaike's Information Criterion.  **P*<0.05.  ***P*<0.01.  ****P*<0.001.  *Source*: IPUMS National Health Interview Survey, 2010-2018, N = 15,912 | | | | |
